# How to tackle the conundrum of quality appraisal in systematic reviews of normative literature/information? Analysing the problems of three possible strategies (translation of a German paper)

**DOI:** 10.1186/s12910-019-0423-5

**Published:** 2019-11-14

**Authors:** Marcel Mertz

**Affiliations:** Working Group Research/Public Health Ethics & Methodology, Institute for History, Ethics and Philosophy of Medicine, Hannover Medical School, Carl-Neuberg-Str. 1, D-30625 Hannover, Germany

**Keywords:** Systematic review, Normative literature, Normative information, Bioethics, Medical ethics, Evidence-based ethics, Methodology, Quality appraisal

## Abstract

**Background:**

In the last years, there has been an increase in publication of systematic reviews of normative (“argument-based”) literature or of normative information (such as ethical issues) in bioethics. The aim of a systematic review is to search, select, analyse and synthesise literature in a transparent and systematic way in order to provide a comprehensive and unbiased overview of the information sought, predominantly as a basis for informed decision-making in health care. Traditionally, one part of the procedure when conducting a systematic review is an appraisal of the quality of the literature that could be included.

**Main text:**

However, while there are established methods and standards for appraising e.g. clinical studies or other empirical research, quality appraisal of normative literature (or normative information) in the context of a systematic review is still rather a conundrum – not only is it unclear how it could or should be done, but also the question whether it necessarily must be done is not settled yet. Based on a pragmatic definition of “normative literature” as well as on a typology of different types of systematic reviews of normative literature/information, this paper identifies and critically discusses three possible strategies of conducting quality appraisal.

**Conclusions:**

The paper will argue that none of the three strategies is able to provide a general and satisfying solution to the problems associated with quality appraisal of normative literature/information. Still, the discussion of the three strategies allows outlining minimal conditions that elaborated strategies have to meet in future, and facilitates sketching a theoretically and practically promising strategy.

## Background

*(This paper is a translated and slightly modified version of: Mertz M. Qualitätsbewertung in systematischen Übersichtsarbeiten normativer Literatur. Eine Problemanalyse. Z Evid Fortbild Qual Gesundhwes 2017;127–128:11–20; the original title and abstract were altered for the translated version.)*

It is more or less impossible to conceive of modern healthcare without (concomitant) reflections upon its norms and ethics. Despite the increased focus on evidence-based healthcare, however, normative and ethical considerations that aim to and indeed should support decision-making and regulation efforts in healthcare practice often still appear to be “eminence-based”. Thus manuals on the creation of guidelines and health technology assessment (HTA) reports, for example, often lack suggestions on how to integrate normative and ethical considerations or even how to capture them in the first place (e.g. [1–3]). However, evidence-based decision-making requires as its foundation – besides clinical experience/internal evidence and patient preferences – systematically collated, comprehensive and balanced external information and external evidence (e.g. from clinical trials or healthcare research) to scientifically justify the decisions taken (cf. [4]). *Systematic reviews* (referred to as “SR” in the following) constitute such a foundation.

For information searched for on specific topics, the SR method aims to identify published studies (*search*), select relevant studies of sufficient quality (*selection*), evaluate these (*analysis*) and summarize or synthesise the individual findings (*synthesis*) (cf. [5] for a similar method of procedure) in a transparent and reproducible way. This method endeavours to guarantee the completeness of the information collected and minimize the risk of bias. Furthermore, this method includes quality weightings of each individual publication in its overall results.

Medical ethics or bioethics [6, 7], the academic field that engages in such normative and ethical considerations, is rooted in the humanities – that is, in theological and philosophical tradition among others – and hence is less familiar with the SR method (cf. [8]). However, due to its interdisciplinary nature, the profile of medical ethics is constantly changing. Formerly, the field’s roots in the ethos of medical practitioners, theology, philosophy and law meant that a philosophical approach to research and clinical judgement played an important role in medical practice. In recent times, however, the profile of medical ethics has changed following the increased integration of empirical insights and research methods taken from the social sciences (see [9–13] and many more).

Alongside the spread of evidence-based medicine (EBM), it is probably these developments that have led to the gradual embracing of SR methods to respond to ethical issues in clinical [14–16] or research [17–19] contexts. While SRs are by no means as widely spread in medical ethics as in EBM, they are no longer merely a niche phenomenon. Evidence shows that the number of SRs is increasing both in medical ethics and in technology assessment, for example as part of research accompanying ELSA (Ethical, Legal, and Social Aspects) or HTA projects. A recently conducted (meta-)SR showed that in the last two decades (1997–2015), more than 180 reviews were published on ethical topics in the medical field [20]. Of these reviews, almost half (47%, *n* = 84) analysed *normative literature* either exclusively or in addition to empirical literature (such as social-scientific studies). Here, normative literature refers to literature that includes normative and ethical considerations, such as ethical arguments, concepts, principles or recommendations [20].

### Definition of the problem

Such SRs of normative literature are carried out based upon a distinct methodological repertoire that ranges from literature search and selection strategies to qualitative and occasionally quantitative methods of analysis and synthesis (cf. [20, 21]). In contrast to SRs in medicine or generally empirical disciplines (e.g. [22, 23]), there are currently no accepted guidelines or manuals on how to carry out SRs of normative literature, but only some published suggestions (e.g. [8, 24–28]). For this reason, only unsatisfactory answers have been provided to methodological questions on how to conduct SRs of normative literature thus far. One of these questions concerns the so-called *quality/critical appraisal* of the literature to be included, which constitutes a key element of (“traditional”) SRs. In such appraisals, the credibility of a clinical intervention study, for example, is judged following an evaluation first and foremost of its *internal validity*, that is, of whether the study design and the actual conducting of the study are able to ensure that the findings are indeed accurate for the patients studied.^1^ In particular, the appraisal critically examines whether a *bias* or a *confounder* that would (statistically) distort the result can be excluded [29].

The results of the quality appraisal have a significant influence upon the weighting of the information collected in regard to the overall synthesis. Only studies that meet methodological minimum or adequacy standards should be included in an SR. Furthermore, higher-quality studies are given a higher weighting, so that in the ideal case the SR’s results are “better” than the results of individual studies. Accordingly, an SR of normative literature needs to state explicitly how the step of quality appraisal is to be dealt with.

Overall, however, the suggestions for SRs of normative literature made to date fail to analyse precisely problems regarding quality appraisal, even though quality appraisal is identified as a challenge (e.g. in [26, 27]). Thus it is hardly surprising that only 24% (20 of 84) of the reviews in the abovementioned (meta-)SR on reviews of normative literature take the step of quality appraisal into account [21]. Five of these 20 reviews even *explicitly refrain* from conducting a quality appraisal, citing *a lack of suitable or applicable methods or criteria for a quality appraisal of normative literature* [21].

The present paper aims to tackle the problem of the criteria and methods required to make a quality appraisal of normative literature – or, more precisely: of normative *information* – within an SR on ethical topics possible. As a first step, a justification of SRs of normative literature is provided. Also, “normative literature” and “normative information”, respectively, are defined more precisely. Next, possible types of SRs of normative literature/information are presented. Three possible solution strategies, the options they offer and their limitations are discussed by reference to, among other things, sample suggestions for quality appraisals made thus far. Subsequently, the paper systematically identifies where, how and by whom quality appraisals can be carried out before concluding by discussing which solution strategy is most suitable to be pursued further.

## Main text

### SRs of normative literature/information

#### Justification and aims of SRs of normative literature/information

Not only statistical information, but normative considerations are important if decisions in healthcare are to be as informed as possible. Accordingly, the value of SRs of normative literature/information consists of their ability to provide comprehensive and transparently collated normative information, for example on a) all known ethical challenges (e.g.: lack of clarity regarding the values or principles relevant to the situation in question; dilemmas; conflicts between norms or principles; weighing of needs) involved in acts undertaken in a clinical or practical research context when using a new (bio-)technology or health intervention (e.g. genome editing processes or informing a patient or relative about a dementia diagnosis), b) all positive and negative arguments that are important for a particular medical decision (e.g. for or against medically assisted suicide), or c) concepts of normative significance in research and clinical practice (e.g. concepts of informed consent in biobank research). Some SRs address ethics specialists, while others aim to support decision-making processes and address persons working in clinics or groups developing guidelines, for example.

Regardless of their target audience, SRs aim to guarantee that all (legitimate) concepts, arguments and challenges are “on the table” at the beginning of a decision-making process and are only set aside once they have been weighed up rationally.

#### Normative literature/information

In light of this background, it is hardly surprising that “normative literature” is often also referred to as “argument-based” or “reason-based” literature (thus e.g. [16, 25, 28]). While such designations intuitively seem to make sense to distinguish this literature from empirical literature (e.g. a social-scientific or clinical study), this differentiation fails to convince in conceptual terms: after all, empirical literature also includes arguments and reasons, as empirical evidence serves to support conclusions or premises that in turn are supposed to support a conclusion [30].

However, it is not easy to come up with a fully convincing theoretical definition of “normative literature”. While there appears to be a certain intuitive agreement on the *scope* of the concept – that is, what kinds of publication it does and does *not* refer to – as the designations “argument-based” and “reason-based” and the results of the meta-SR [20, 21] suggest, the *intension* of the expression – the definition of the characteristic traits of what the concept refers to – is much more difficult to grasp. For this reason, the definition suggested in the following has a pragmatic claim: it aims to include what has been discussed (hitherto) in the debate on SRs of normative literature/information; and it aims to do justice to the non-empirical (or not wholly empirical) literature identified and evaluated in the SRs carried out to date in medical ethics (cf. [20, 21]), that is, to make it possible to distinguish between different types of SRs of normative literature/information (see *Types of SRs of normative literature/information* below). Therefore the definition will not be able to exclude every possible grey area:

*Normative literature is literature that (i) aims to evaluate judgements, decisions, acts, (social) practices, technologies, institutions, organizations and general states of the world from a moral or legal point of view and/or to define/set out which decision or course of action is or should be morally or legally necessary, prohibited or permitted; or that (ii) aims to develop, interpret or criticize the evaluative or prescriptive concepts required for this.*

To render this more precise, it should be noted that “decisions” can also include regulations (policy), and “judgements” can also include reasons for or against decisions. “Concepts” also includes values, norms, principles and theoretical frameworks; the enumeration at the beginning of the definition (judgements, decisions...) should not be understood as conclusive.

“Empirical literature”, on the other hand, does not serve the purpose of evaluation but rather (only) of description, explanation and prediction. The fact that empirical information may be necessary for an evaluation plays no role in this: the literature’s aim is decisive, not the way in which the findings of the literature (e.g. of a clinical trial) may or have to be utilized. However, some information found in empirical literature can, in principle, be used as normative information, e.g. perspectives on a specific ethical dilemma in praxis that were elicited by an interview study. In this regard, at least some parts of empirical literature can be included in SRs of normative literature/information.

The definition provided above takes into account jurisprudential and legal literature insofar as it can be of ethical relevance. Of course it can also be seen as a type of normative literature in its own right. As this essay is concerned primarily with normative *ethical* literature and information, however, legal literature will only be touched upon in passing.

### Types of SRs of normative literature/information

Different types of SRs of normative literature/information can be distinguished based upon their different purposes and units of analysis, i.e. based upon the normative information that is extracted from the literature. This typology is important as the kind of normative information searched for has significant implications for the question of how normative literature – or the information itself – can be appraised. Taking a typology developed by McDougall ([8], p. 91), the examples mentioned above and the findings of the (meta-)SR on overviews of normative literature [20, 21] as a starting point, one can distinguish between at least six different types, listed in Table 1 below:

**Table 1:** Types of SRs of normative literature/normative information

Type	Explanations	Examples in the literature
SRs of ethical conclusions	Only the “all things considered” conclusions of ethically relevant arguments on a certain topic (e.g. on concealing the administration of drugs)	[25, 31]
SRs of ethical arguments	Ethically relevant arguments (or reasons) concerning a certain topic (e.g. whether post-trial access is a moral requirement in drug trials)	[15, 17]
SRs of ethical issues	Ethical issues to be considered, e.g. when treating medical conditions (e.g. dementia) or when using new technologies (e.g. assistive technologies for elderly patients)	[33]
SRs of ethical concepts	Ethically relevant definitions (e.g. “moral distress”) and concepts/approaches (e.g. “nursing ethics”)	[16, 35]
SRs of ethical values/norms/principles/	Values, norms and principles concerning certain possible courses of action and (clinical) fields of action (e.g. in plastic surgery)	[36, 37]
SRs of ethical recommendations	Recommendations from guidelines, handouts, commentaries issued by ethics councils/ethics commissions etc. (e.g. on the question of whether underage persons can be living donors)	[38, 39]

According to Strech et al. (e.g. [32, 33]), “ethical issues” in *SRs of ethical issues* are defined in such a way that an ethical issue is present when either one or several principles are not considered in an action situation or a decision-making process (even though they should be considered), or when there is a conflict between two or more principles and they thus need to be weighed against one another. The four-principle approach is taken as a starting point. This approach is well established in medical ethics and formulates four principles of “medium” scope (as keywords: *respect for patient autonomy*, *non-maleficence*, *beneficence*, and *justice*). More generally, “ethical issues” cover what was described above under “ethical challenges”, such as a lack of clarity concerning relevant values or principles, the existence of dilemmas or situations in which needs need to be weighed up in general (also cf. [20]).

Some of the SRs listed in Table 1 may also use empirical literature, but here they are only viewed from the perspective of their use for finding normative information. McDougall’s typology also includes further SRs of empirical literature in medical ethics that are not listed here. When considering jurisprudential and legal literature as normative literature, *SRs of legal norms* (e.g. national or international laws and regulations), *SRs of case law* (e.g. of state or federal courts) or *SRs of legal commentaries* (e.g. on legal norms or on case law) are conceivable. However, this paper will not go into these possible types in greater detail due to its focus upon SRs of normative ethical literature/information and due to the lack of actual examples of such SRs.

As (ethical) *arguments* can be of significance in one way or another in most kinds of normative literature, they will be given particular emphasis in the following section and used as an example.

### Solution strategies for the quality appraisal of normative literature/information

As already mentioned above, at present there are no standards for the quality appraisal of normative literature or information within SRs. For this reason, the solution attempts that currently exist and those that are conceivable for the problem of quality appraisal are summarized in three strategies: firstly, *appraisal using (global) reporting guidelines*, secondly *appraisal using (procedural) quality assurance guidelines* or the *decision to forego an independent appraisal*, and thirdly the *appraisal using content-related quality criteria*. Only the latter strategy will focus decidedly on the normative information itself, while the other strategies are focussing more on appraising a piece of literature.

### Solution strategy 1: appraisal using (global) reporting criteria

The first solution strategy follows the idea of reporting guidelines (see [40]) and the criteria derived from them that are usually implemented in the form of checklists. Here, the text from the literature (e.g. journal article) is appraised *as a whole* (is the article’s reporting of sufficient quality or not?). The McCullough model of an *SR of ethical conclusions* [25] serves as an example of this strategy, pursuing the aim of formulating an ethical recommendation for action on the basis of the normative information found. The model suggests various criteria that can be gone through like a checklist and that evaluate the following domains ([25], p. 69): whether the publication has used a focused question; whether the publication has searched for literature and how clear an account it gives of the literature; the quality of the ethical analysis and arguments; the clear description of the conclusion; and the clear specification of the clinical application of the ethical analysis, argumentation and conclusion. The domains were developed according to the authors’ earlier suggestions for appraising “argument-based ethics” ([26]; for comparable criteria see [41, 42]). Each domain is evaluated using a “scoring system” either as “0″ (criteria are not met), “1″ (criteria are met in their entirety) or “1/2″ (criteria are partially met).

The McCullough model was strongly criticized, especially by Sofaer and Strech [27]. Criticism targeted its overly subjective and random scoring system in particular, although the authors themselves had already admitted this was a limitation ([25], p. 72). However, the scoring system is by no means compulsory for this first solution strategy, which is why the following subsections will examine those problems that generally impede appraisals that use reporting criteria:

#### One-sidedness

A “catalogue” of criteria such as that of McCullough et al. becomes one-sided as soon as it is used to evaluate not only philosophical literature in a narrower sense, but, for example, guidelines or legal literature (cf. [27]), as these kinds of texts can differ markedly in regard to their methodology and the accounts given. Furthermore, such reporting criteria are often formulated according to the publication standards of “analytical” philosophy, or analogously to the empirical (social) sciences, and thus place the contributions of “continental” philosophy or the other humanities at a disadvantage. Hence a tendency towards distortion becomes part of the evaluation, as “continental” philosophical contributions are less able to meet the criteria due to the different reporting conventions.

Such reporting criteria are designed not least for journal articles, and are not really applicable to all contributions in edited books and certainly not to whole monographs. While the latter are not covered in “traditional” medical SRs, there are good reasons for considering them in SRs of normative literature [32].

#### Lack of differentiation in global appraisals

One aim of the first solution strategy is to evaluate a text’s quality of reporting as a whole in order to decide whether to include or exclude it on this basis. This global appraisal is based upon the implicit premise that a text’s (reporting) quality says something about the quality of the (individual pieces of) information *as a whole*. However, this premise is more plausible when applied to empirical literature (especially clinical trials) than to normative literature: an empirical study’s reporting is key to reviewing and verifying the quality of the data and their analysis. In the best case, the reporting corresponds to the trial actually carried out; therefore, it is (more) justified to define the quality of the trial’s results based on the article’s reporting quality. Where normative literature is concerned, however, this kind of interrelation is present to a much lesser extent, for these texts may contain several pieces of normative information (or arguments) – both those *endorsed* as well as those *criticized* [27]. Furthermore, an article that by and large fulfils the criteria of good reporting (e.g. “uses a focused question” or “clear account given of the conclusion”) may nevertheless be questionable in terms of its content, containing weak arguments or making ethically dubious recommendations, for example. Accordingly, we cannot conclude from “poor compliance with reporting criteria” that the “concrete content of the literature is of poor quality”. More decisively, an article that does not fully meet reporting criteria can nevertheless be significant in terms of its content (e.g. presenting a good argument). Ultimately, not including normative information in the synthesis of an SR of normative literature because the text is not of high (reporting) quality overall would lead to a distorted selection of normative information and achieve precisely the opposite of that which quality appraisal is supposed to ensure. This procedure is conceivable at best in *SRs of ethical conclusions*.

#### Possible mixing of reporting criteria and content-related quality criteria

McCullough et al. also include a criterion about the quality of the ethical analysis and the arguments in their catalogue, possibly in order to mitigate the problem discussed above. However, this criterion differs markedly from the other reporting criteria, and hence it is doubtful whether such a criterion is consistent with the other criteria – after all, reporting criteria only evaluate whether something has been mentioned sufficiently frequently, not the quality of what is reported. Furthermore, given that the reporting is appraised as a whole, this criterion is not sufficiently differentiated in terms of content to be able to make reliable statements on the quality of concrete content. However, if the criterion were differentiated further and operationalized more precisely, for example to examine a text’s individual arguments, it would no longer correspond to a reporting criterion, but would instead merge with the content-related quality criteria of solution strategy 3 examined in greater depth below. Finally, the question of whether this criterion would be able to balance out possible flaws in adhering to the reporting criteria would require further clarification.

#### Conclusion solution strategy 1

The first solution strategy can at best be applied to *SRs of ethical conclusions* that focus mainly on a text’s “all things considered” conclusion (as in the McCullough model) – provided the normative text to be evaluated discusses only one single line of argument and thus arrives at a definite conclusion. The abovementioned problem of one-sidedness could be mitigated by developing a comprehensive catalogue of criteria that takes account of different kinds of normative literature and different styles in a differentiated manner. However, as soon as the aim is to review the quality of individual pieces of information (among other things), for example in *SRs of ethical arguments* or *SRs of ethical issues*, reporting criteria and the global appraisal that goes hand in hand with them are no longer differentiated enough. Even if the quality of analysis and argumentation is (also) considered – which is already tantamount to a content-focused appraisal procedure, however – a *blanket* appraisal such as that of McCullough et al. fails to clarify how the hitherto unresolved question of how to evaluate the content of normative literature is to be dealt with. For example, the question of how a valid deductive argument with false premises should be evaluated ([27], p. 319) needs to be resolved.^2^ This is aggravated by the fact that in inductive (e.g. probability conclusion) and abductive forms of argument (e.g. “conclusion to the best explanation”), which also occur in medical ethics, validity is an irrelevant criterion: there are no (formal) logical rules of conclusion (however, e.g. statistical considerations may play a role), which is why we need to speak not of validity, but of *strength* or *explanatory power*. Furthermore, we simply do not always know whether an argument’s (empirical) premises are true or not. This can itself form the subject of current scientific debate. Not least, the appropriateness of normative and evaluative premises is an excellent topic of rational debate. It is true that solution strategy 3, discussed below, will also face these issues; however, a criterion along the lines of “quality of argumentation in this article; good – not good?” to be “checked off” from a reporting-criteria-style checklist will not be able to provide any answers to these questions, and therefore the appraisal will contain comparatively high levels of subjectivity. While the latter can be somewhat mitigated by, for example, a method characterized by a consensus-driven approach in which different researchers carry out their evaluation independently of one another and then compare their results, it cannot be avoided entirely. Finally, SRs using normative information from (also) empirical literature is not well-considered in this strategy, as it is focussing solely on (a piece of) normative literature as a whole.

### Solution strategy 2: appraisal using (procedural) quality assurance criteria

McDougall, who drew upon McCullough’s model for her own *SR of ethical conclusions* [26, 31], finally rejected this model because of the abovementioned problems with this quality appraisal method and instead fell back upon using criteria associated with the characteristics of a primarily procedural *quality assurance* of a text: if an article was published in a journal that uses peer review, or was published as a book chapter in a volume printed by a “prominent academic publisher”, it is assumed that its quality is sufficient ([26], p. 95). Accordingly, the peer review process and the academic publishers’ reputation, which is assumed to be based (among other things) on their quality assurance, thus served as a criterion. McDougall explicitly chose not to evaluate the literature further.

The Strech-et-al. model of *SRs of ethical issues* [14, 32, 33] also falls back upon such quality assurance criteria. These SRs aim to cover as comprehensive a range of ethical issues concerning a medical condition and its treatment as possible. They do not aim to make any recommendations for dealing with these issues. Here, quality assurance criteria are dealt with as part of the criteria for inclusion or exclusion and not as a separate step in the selection process. Publication in a peer-reviewed journal or a serious academic book publication likewise serves as a quality assurance criterion; in addition, “national-level reports” are seen to assure quality ([32], p. 401; similarly [14], p. 202–203; [33], p. 7).

The selection of procedural quality assurance criteria and the foregoing of any quality appraisal beyond this are justified with the descriptive aim of generating as comprehensive a spectrum of the ethical issues in question as possible; the literature’s quality is not relevant to achieving this aim ([33], p. 7). The authors of the Sofaer-Strech model for *SRs of ethical arguments* [27, 28] argue in a similar vein. This model likewise has a descriptive aim, namely a comprehensive representation of all reasons (arguments) concerning an ethical issue. Because of this aim, it is not necessary to evaluate the quality of the literature ([27], p. 320). However, the authors do acknowledge that this procedure is owed to a certain pragmatism and the difficulty of developing quality assurance criteria for normative literature ([27], p. 324f).

Usually, peer review and similar processes cannot be used as quality assurance criteria for *SRs of ethical recommendations*. However, the reputation, (e.g. political) legitimation or the (disclosed) methods of the respective organization (e.g. expert association) take on a comparable function when generating recommendations.

Nevertheless, this solution strategy is not without its problems:

#### The limitations of the peer review process and other quality controls

Quality assurance through peer review has its limits, some of which are considerable [43, 44]. For this reason, it is a rather weak criterion for the quality of normative literature. Furthermore, it hardly makes sense that only articles to have gone through peer review and high-ranking book publications should be included if it is claimed that quality appraisal plays no role in the respective aim of the SRs of normative literature – after all, normative information such as arguments or significant issues can also be found in “grey” literature or less high-ranking book publications (e.g. self-published books). Accordingly, a more convincing approach is to use this criterion as one of several criteria for inclusion or exclusion that determine which texts are selected from the hypothetical total amount of relevant literature. The justification for this is pragmatic (reducing the amount of literature to be viewed while remaining transparent concerning the search procedure), or is simply restricted to increased “epistemic trust” arising from the fact that the literature included has gone through at least a certain form of quality assurance. However, even then no direct conclusions can be drawn concerning the quality of *individual* pieces of normative information.

#### Aim-dependency

The argument that an independent quality appraisal can be eschewed depending on the SR’s aim and that referring to the publication medium’s quality assurance is sufficient is only convincing in the case of SRs of normative literature with a highly descriptive orientation. Of course, in the first instance all SRs of normative literature are necessarily descriptive in regard to their findings, that is, their summary of normative information. Only the kind of synthesis decides whether normative conclusions are drawn from the descriptive compilation of normative information. However, not all SRs of normative literature pursue an exclusively descriptive aim. For this reason, choosing not to carry out an independent quality appraisal can be justified only in the case of individual SRs of normative literature on the basis of their questions or more generally for particular kinds of SRs of normative literature, not for SRs of normative literature in general. This restricts the scope of this solution strategy markedly – unless one argues that SRs of normative literature are only allowed to pursue *exclusively* descriptive aims.

#### Problematic content quality

Apart from the possible charge that purely descriptive aims fail to fulfil the mandate of medical ethics as a normative endeavour – such as assessing what particular normative information means for ethical decision-making or for formulating recommendations and so on – the importance of the quality of the information compiled for an SR of normative literature is downplayed unjustly. This is particularly the case when the evaluation of the information is left up to the users of SRs of normative literature, who may not be familiar with medical ethics. For example, arguments can be one-sided, somewhat implausible or even draw fallacious conclusions, but the users of SRs of normative literature may not recognize this in some circumstances. The same also applies to ethical issues. An ethical issue in the literature could be insufficiently theoretically or empirically grounded or simply be irrelevant (also see solution strategy 3).

#### Conclusion solution strategy 2

Procedural quality assurance criteria may be admissible as a minimum standard from a pragmatic point of view. In the case of *SRs of ethical issues, SRs of ethical concepts* and *SRs of ethical norms* in particular, referring to these criteria can be seen as sufficient, as here it is easy to justify that their sole aim is to describe the topics, norms and so on occurring and discussed in scientific literature. Much the same goes for *SRs of ethical recommendations*. It is also an advantage of this strategy that it can encompass empirical literature as a source of normative information, as a criteria such as “being peer-reviewed” is quite independent of the type of literature. However, quality assurance criteria are not a convincing solution to the problem of quality appraisal in general, as can be seen particularly clearly in the case of *SRs of ethical conclusions* and *SRs of ethical arguments*, which do not pursue purely descriptive goals but aim to support ethically appropriate decision-making. In any case, leaving an independent evaluation of quality out without giving reasons for doing so is not legitimate, and will remain so until there are recognized methodological standards that render a quality appraisal unnecessary (and that authors can explicitly refer to). The fact that the type of SR of normative literature/information in question does not depend on the quality of the text or the normative information it contains is a legitimate reason to forego an appraisal. As argued above, however, it is not always easy to prove that this is the case.

### Solution strategy 3: appraisal using content-related quality criteria

The third solution strategy was already mentioned in the discussion of the first strategy: criteria that refer directly to the quality of the content of the normative *information* in question (e.g. arguments or issues) are employed in the appraisal. Here, existing criteria and methods from the field of informal and formal logic, rhetoric, critical thinking and philosophy in general (e.g. [45–47] and many more) can be drawn upon. For example, the evaluative criteria for deductive arguments (validity and soundness), inductive arguments (strength) and abductive arguments (explanatory power) as well as the respective operationalizations for subtypes of inductive arguments in particular (e.g. inductive generalization, statistical syllogism or analogy conclusion) can be used (cf. [46]). Likewise, criteria for certain types of definition can be formulated using definition theory. Especially the ample literature on informal fallacies, together with knowledge of typical sources of cognitive bias (e.g. heuristics) can be used to pose critical questions, including those concerning the production of normative information. Various well-known rebuttal and criticism strategies (such as counterexamples, reductio ad absurdum, horned dilemma etc.) could also be transformed into suitable questions or be operationalized in criteria. Thus the focus could shift to the semantics, truth and plausibility as well as the justification of premises.^3^

Thus far, however, there are no concrete models of a quality appraisal method for SRs of normative literature/information that this solution strategy could outline. For this reason, the possibilities and challenges of this strategy will be explored cursorily here:

#### Position of the appraisal within the methodological process

In “traditional” SRs, quality appraisal usually forms part of the *selection* step (selecting the texts). In solution strategy 3, it scarcely seems to make sense to include the evaluation of quality in the selection stage. Individual pieces of normative information need to be first identified in order to be evaluated, which is part of the *analysis* step; but for this step, texts already need to have been selected. The appraisal would then determine whether normative information is actually extracted, that is, whether it becomes part of the analysis of an SR or not. However, appraisal would not (or no longer) decide whether a given *text* is included or not.

Likewise, undertaking the evaluation only in the final *synthesis* step is also conceivable (suggested in [27], p. 324–325): the synthesis of normative information, such as ethical arguments, would only include those items of information for which a certain level of quality could be vouchsafed. Furthermore, the respective appraisal of quality can be noted transparently for each information (e.g. argument) or its summary.

#### Implementing existing criteria in appraising normative information

There is not really a need for completely new criteria or methods to evaluate normative information. However, applying these criteria and methods in a time-efficient and objective manner appears to be difficult, even though an *SR of ethical arguments* with a normative aim, for example, really would require such an elaborate appraisal. For example, making statements about an argument’s quality would usually necessitate a reconstruction, analysis and criticism of its structure and content. This requires the researchers carrying out the SR both to possess sufficient knowledge of the abovementioned criteria and, in most cases, be familiar with the content of the argument’s subject. Seminars on informal logic regularly illustrate how time-consuming it can be to reconstruct arguments adequately and evaluate them fairly. One of the reasons for this is the fact that in practice, the question of whether an argument is “good” or not inevitably is a discursive matter: some will agree and list reasons in favour, others will disagree and will likewise offer support for their opinion (thereto, cf. [27]).^4^ Evaluating an argument without presenting another argument on why it is “good” or “bad” will probably tend to be overly subjective. What is missing are practicable, albeit not overly simplified criteria and methods for evaluating especially arguments, but also issues and concepts *within the context* of SRs of normative literature or normative information.

#### Ethical relevance as an attribute of quality

Where the evaluation of the individual items of normative information is concerned, another question of *relevance* arises, namely that of the information’s *ethical* relevance. This needs to be distinguished from the relevance criteria that apply to the selection of literature. The question of the ethical relevance of normative information may become noticeable in the analysis and especially the synthesis stages. In the analysis, because of often more or less implicit evaluations a decision will have to be made on whether information is extracted or not; in the synthesis, the decision concerns whether and how the information is to form part of the summary. That is, instead of extracting *all* arguments from the literature selected, for example, those regarded as irrelevant to the ethical problem at hand will not be extracted – whether because they are simply not applicable thematically (e.g. an argument concerning abortion in an SR on post-trial access), are judged to be secondary on the basis of the values, norms and criteria they use (e.g. a deontological argument when utilitarian criteria are assumed), their empirical premises (if present) are regarded as insufficiently supported or overly speculative, or the actions they demand cannot be (“realistically”) put into practice. Similarly, an ethical issue could also be irrelevant, for example because it hardly ever occurs empirically.^5^ Accordingly, when the aim is to outline ethically relevant arguments or issues, the relevance of the individual items of normative information seems to become a characteristic of quality: irrelevant arguments or aspects would distort the results of an SR. Only SRs fully committed to descriptivity will be able to avoid the question of relevance as part of their quality appraisal. Besides the theoretical identification of ethical relevance with quality, the main problem is the question of which methods (e.g. application of ethical theories?) can be used to define this relevance to limit subjectivity or at least intransparency.

#### Conclusion solution strategy 3

The third solution strategy is probably best able to accommodate the specific traits of normative literature and especially information, and the methodological particularities of corresponding SRs – foremost in the case of *SRs of ethical arguments* or SRs with a (strong) normative aim. Also, this strategy allows for appraising normative information extracted from empirical literature as well. The question of the availability of criteria and methods that are both adapted to the purposes of SRs and practicable remains unanswered – at least for now. (See Table 2 for an overview of the suitability and challenges of the solution strategies.)

**Table 2:** Overview of the suitability of and critical reflections on the solution strategies

Solution strategy	Criteria	Possible suitability	Critical reflections
1	Reporting criteria (predominantly)	SRs of ethical conclusions	• One-sidedness • Lack of differentiation in global appraisals • Possible mixing of reporting criteria and content-related quality criteria
2	(Procedural) quality assurance criteria (or decision not to carry out an independent appraisal)	SRs of ethical issues SRs of ethical concepts SRs of ethical norms SRs of ethical recommendations *Only with descriptive aims:* SRs of ethical conclusions SRs of ethical arguments	• The limitations of the peer review process and other quality controls • Aim-dependency • Problematic content quality
3	Content-related quality criteria	All, but particularly SRs of ethical arguments and SRs with a (strong) normative aim	• Position of the appraisal within the methodological process • Implementing existing criteria in the appraisal of normative information • Ethical relevance as an attribute of quality?

### The “6Qs” of a method for appraising the quality of normative literature

Regardless of the concrete way in which the problems of the three solution strategies are tackled, their discussion has clearly shown not only that is it unclear exactly *how*, using which criteria or method, a quality appraisal is to be carried out, but that not even the question of *what* exactly is to be appraised and *where* within the methodological procedure of an SR of normative literature this appraisal should take place has been clarified.

On the basis of the strategies discussed, the findings of the meta-SR [20, 21] and further theoretical considerations, however, it is possible to define some *minimum conditions* for the design of a quality appraisal method for normative literature. That is, regardless of its respective concrete design, a method needs to make statements on certain aspects if it is to adequately appraise the quality of normative literature. These minimum conditions can be expressed as six questions or “6Qs”, respectively: *what* (the element or information in a text that is evaluated), *which* (which text; genre of the respective text), *where* (in the procedure of carrying out the SR), *how* (using which criteria or methods), *whereby* (the process of applying the criteria or methods) and *who* (which disciplinary or methodological background do the researchers need to have). Table 3 provides an overview with four *sample* answers to these questions, without making any claim to comprehensiveness.

**Table 3:** The “6Qs” of a method for appraising the quality of normative literature

What (Element of evaluation, type of normative information)	
• Individual pieces of normative information (individual conclusion, individual argument, individual concept etc.) • Whole line of argument (may be identical to part or the whole of the text) • Part of the text (paragraph, chapter) • Complete text (article, book chapter)	
Which (Type of text)	
• Normative ethical text (philosophical) • Empirical text • Guideline text • Jurisprudential/legal text	
Where (Methodological procedure/step)	
• As part of the criteria for including or excluding a text (selection) • Separate appraisal following the inclusion of the text • Appraising the units of analysis (analysis) • Appraising the findings (synthesis)	
How (Criteria, method)	
• Explicit decision not to carry out a quality appraisal • Reporting criteria • (Procedural) quality assurance criteria (peer-review process etc.) • Content-related quality criteria (e.g. appraisal of individual arguments)	
Whereby (Process)	
• Individual researcher • Individual researcher reviewed by other researcher • Several researchers in a consensus-driven process • Several researchers with a comparison of their ratings	
Who (Disciplinary/methodological background)	
• Primarily philosophical background • Background primarily in the type of normative literature (e.g. law in legal literature) • Background primarily in systematic review methodology • No specific background (method needs to/can be learned)	

The responses in the categories can be combined in different ways to outline a concrete solution strategy (e.g. “individual piece of normative information (conclusion)” plus “normative ethical text” plus “separate appraisal following inclusion” plus “reporting criteria” plus “individual researcher with review” plus “no specific background”). However, some answers in one category may suggest or exclude answers in another category. For example, if the entire text is to be evaluated, then this appraisal will not be possible in the analysis or synthesis steps. Anyone wanting to use quality assurance criteria will necessarily (also) evaluate the entire text. And those committing themselves to content-related quality criteria will probably need researchers to have a suitable disciplinary/methodological background. This illustrates that the “6Qs” and the sample ways of answering them in Table 3 can serve as a heuristics for developing newer or more refined quality appraisal methods. However, they can also be used analytically to systematically classify existing methods.

## Conclusions

It is probably undisputed that SRs alone are unable to “do medical ethics”. Philosophical or interdisciplinary detailed analyses of the respective ethical challenges, key normative concepts and relevant norms remain necessary. This is affirmed from the perspective of SRs of normative literature themselves: after all, this type of literature forms their subject.

However, SRs of normative literature/information make it possible to search, select, analyse and summarize this literature in as transparent and undistorted a way as possible. As a form of “evidence-based” ethics, they should be understood as part of evidence-based healthcare. Therefore, such SRs are of particular relevance to anyone producing systematically developed decision guidance such as guidelines or HTA reports. Not least, systematic accounts of all ethical arguments made thus far concerning a particular topic, for example, may be of significance for research in medical ethics itself. In this way, arguments less strongly debated in the literature can be given an equal weighting, countering one-sided lines of argument [27]. Accordingly, making methodological contributions to improving the quality of SRs of normative literature/information, especially where the key aspect of quality appraisal is concerned, is all the more important.

But which quality appraisal method seems most promising? It seems obvious that it would be one that is able to provide clear answers to the “6Qs” and that in principle follows solution strategy 3, although it can certainly draw upon complementary criteria from the other two solution strategies. In doing so, however, it needs to do justice to the aims of an SR (descriptive/normative) and the SR type (*SR of ethical arguments, SR of ethical issues* etc.). This *can* include the justified decision to forego an (independent) appraisal of quality. The method would furthermore have to be adaptive, that is, it would need to adapt its appraisal to the different kinds of normative literature (ethical/legal) and to the normative information searched for (argument, issues, concepts...). Finally, content-related quality criteria, for example the evaluation of arguments, should not be applied during the selection, as in “traditional” SRs, but only during the *synthesis*.

Despite its greater complexity, an approach that combines criteria and methods will be more effective than the “one size fits all” approaches suggested and discussed thus far (e.g. [24, 28]).

## Data Availability

Not applicable.

## Abbreviations

EBM: Evidence-based Medicine
ELSA: Ethical, Legal, and Social Aspects
HTA: Health Technology Assessment
SR: Systematic Review(s)

## Footnotes

1 Generalizability and transferability to practice, which are subsumed under external validity, have not been considered to any great extent in quality appraisals thus far (cf. [29]).

2 The validity of a deductive argument is a purely formal trait that exists independently of the premises’ truth or even meaningfulness. An argument is valid if the premises employed necessarily lead to the conclusion given; the content of premises and conclusion is irrelevant. Therefore, a valid argument is not always sound. This is only the case if validity is present and true premises have been used.

3 Content-related quality criteria could thus be defined both by their content in a narrower sense (e.g. “Is an argument’s premise true or at least plausibly acceptable?”) and in formal (logical) terms (e.g. “Is a deductive argument valid?”). Both can be key to the quality of the content of a normative information item and are thus subsumed under “content-related quality criteria”, especially in distinction to the reporting and quality assurance criteria discussed above.

4 It is not possible to answer the meta-ethical question of whether it is even philosophically possible in principle to define objective criteria for the quality of normative information that go beyond e.g. the logical validity of deductive arguments, especially as a large number of mainly epistemological presuppositions are decisive for this – for example, whether cognitivism is assumed (including the assumption that normative propositions such as norms can be true in the same way as empirical propositions) or non-cognitivism (including the assumption that normative propositions cannot be true and only express that speaker’s feelings, for example). As the present author assumes that discourse is inevitable in the (interdisciplinary) practice of a scholarly or scientific method (such as an SR), ultimately the aim of a method for appraising the quality of normative literature can only be to develop critically reviewed and grounded criteria and methods that are able to create intersubjective consensus and that those carrying out such SRs are able to put into practice. Anyone rejecting as a matter of principle the possibility of criteria that are pragmatic and able to achieve intersubjective consensus will probably have little interest in carrying out SRs of normative literature. However, the present essay is not written for those espousing such positions, as it assumes that criteria that are pragmatic and able to achieve intersubjective consensus are fundamentally possible.

5 Whether an issue occurs empirically or not, or whether an empirical premise in an ethical argument is true or well founded, requires pertinent empirical literature. The extent to which the empirical occurrence of ethical issues or the (sufficient/insufficient) support of empirical premises can or should be used to determine ethical relevance is a meta-ethical question that cannot be dealt with within the scope of this paper.
